# Navigating the Ghanaian health system: stories from families of children with intellectual and developmental disabilities

**DOI:** 10.1080/20473869.2020.1865121

**Published:** 2021-01-08

**Authors:** De-Lawrence Lamptey

**Affiliations:** School of Rehabilitation Therapy, Queen’s University, Kingston, ON, Canada

**Keywords:** Ghana, global child health, intellectual and developmental disabilities, pediatrics, interpretive description, healthcare delivery

## Abstract

This study explored the experiences of families in navigating the Ghanaian health system to address the general health needs of their children with intellectual and developmental disabilities (IDD). The sample involved 22 primary caregivers of children with IDD aged 3-18 years who participated in a semi-structured interview. The interviews were analyzed using the constant comparison analytical method. The findings highlighted key enablers and barriers related to three overarching themes: entry into the health system; consultation with health professionals; and service coordination. The findings showed that the families and their children gained entry into the health system in many health facilities. However, the families revealed that some facilities denied the children services, either because the children had difficulties following entry processing protocols or there were no health professionals willing to address the children’s needs. Although health professionals perform their duties professionally during consultation and care administration in many cases, the families reported on some challenges. Service coordination was seamless in some facilities; however, the families reported on other facilities they accessed where service coordination was not seamless. The study findings illustrate that the experiences of families and their children with IDD in the Ghanaian health system may be mixed.

## Introduction

The incidence of many health conditions, such as respiratory, oral, gastrointestinal, sensory, cardiovascular, neurological and endocrine conditions, can be relatively high in children with intellectual and developmental disabilities (IDD) (Allerton *et al*. 2011, Määttä *et al*. 2011, Oeseburg *et al*. 2011, Schieve *et al*. 2012, Taggart and Cousins 2014). Although many of the health conditions that can affect children with IDD can also affect typically developing children, children with IDD are more vulnerable due to the etiologies of IDD. The etiologies of IDD may be prenatal (e.g. genetic/chromosomal abnormalities), perinatal (e.g. birth injuries) and postnatal (e.g. childhood infections and brain injuries) (American Psychiatric Association 2013, McDermott *et al*. 2007, Wehmeyer *et al*. 2017). These etiologies may negatively impact the development and functions of body organs and systems to induce health conditions (McDermott *et al*. 2007, O’Hara *et al*. 2010, Taggart and Cousins 2014). Side effects of medications used in managing neurological and behavioral problems that may be associated with IDD may also potentially contribute to health issues (Aronson 2015, Gardner and Teehan 2010, McQuire *et al*. 2015). Further, receptive and expressive language challenges associated with IDD (American Psychiatric Association 2013) may have a negative impact on the ability of the children to articulate health symptoms to their families or health providers for timely responses.

Despite the physiological and neurological predispositions of children with IDD to health issues, the literature highlights key barriers that impede healthcare for children with IDD in many parts of the world. For example, studies show that families of children with IDD in the United States, Turkey, India and Pakistan may encounter financial barriers to healthcare (Juneja *et al*. 2012, Mirza *et al*. 2009, Saunders *et al*. 2015, Schieve *et al*. 2012). As well, children with IDD in rural communities in Canada, Australia, Pakistan and the West Bank may be confronted with geographic disparity of health services (Dababnah and Bulson 2015, Hussain and Tait 2015, Kreitzer *et al*. 2016, Mirza *et al*. 2009). Geographic disparity in Canada may lead to sporadic services for children with IDD in rural communities (Kreitzer *et al*. 2016). In Australia, Pakistan and the West Bank, geographic disparity could mean that families in rural communities or areas affected by territorial disputes may have to travel elsewhere to access services for their children (Dababnah and Bulson 2015, Hussain and Tait 2015, Mirza *et al*. 2009), which may heighten the financial burden on families or impede access to timely healthcare. Children with IDD in the United Kingdom and the United States may experience higher delays in the health system compared to the general population (Heslop *et al*. 2014, Schieve *et al*. 2012), which may highlight unequal access for children with IDD. Lim *et al*. (2012) also illustrate that children with IDD in China may experience delays in the health system because of shortage of health professionals to meet high volumes of service demand.

Many people in the general population may encounter many of the barriers that impede healthcare for children with IDD. However, the increased health needs that may be induced by the physiological and neurological conditions associated with IDD may aggravate the rate at which children with IDD encounter these barriers. While the contributions of physiological and neurological conditions to the health needs of children with IDD may be relatively inevitable, the barriers that impeded healthcare for the children can often be avoided through social actions that promote equitable healthcare for all (e.g. universal health coverage) (Marmot 2013, United Nations 2015, World Health Organization 2015).

Contrary to the considerable literature on the health needs and experiences of children with IDD in accessing healthcare in many parts of the world, two recent scoping reviews on health and rehabilitation services for children with disabilities in low-and middle-income countries demonstrate that relatively little is known about the needs and experiences of children with IDD in Ghana (Adugna *et al*. 2020, Magnusson *et al*. 2019). Research linked to healthcare for children with IDD in Ghana have largely focused on screening and diagnosis of IDD (Bello *et al*. 2013, Bornstein and Hendricks 2013, Gottlieb *et al*. 2009, Kyeremateng *et al*. 2019), health beliefs and behavior of families toward the needs of their children with IDD (Lamptey 2019) and attitudes of health professionals toward children with IDD and their families (Oti-Boadi 2017). For example, Bello *et al*.’s (2013) study on screening for developmental delays among children attending a rural community clinic suggests that children who were identified as potentially having developmental delays in this rural community had access to healthcare to an extent. However, aside from disability screening, Bello *et al*. (2013) did not explore further health needs the children may have had, and how those needs may have been addressed at the rural community clinic. Kyeremateng *et al*. (2019) also highlight the plight of families from other regions in Ghana who travelled to Accra, Ghana’s capital, to seek formal diagnosis and care for their children’s developmental delays and disabilities. While families acknowledged that they received support from health providers to an extent, the families highlight that they also experienced negative attitudes from providers (Oti-Boadi 2017).

Although the emerging studies from Ghana provide some insight on the experiences of children with IDD in the Ghanaian health system, evidence suggests that researchers have paid little attention to how the Ghanaian health system addresses the general health needs of children with IDD beyond disability screening and diagnosis. This research gap is unfortunate, particularly since research from around the world illustrates that the incidence of many health conditions can be high in children with IDD (Allerton *et al*. 2011, O’Hara *et al*. 2010, Oeseburg *et al*. 2011, Schieve *et al*. 2012, Taggart and Cousins 2014). A deeper understanding of the general needs and experiences of children with IDD in Ghana can provide policymakers, service providers and health administrators with important directions for improving services for the children. The purpose of this study was to explore the experiences of families in navigating the Ghanaian health system to address the general needs of their children beyond seeking a diagnosis of IDD. This study is part of a large ongoing research exploring the health needs and experiences of children with IDD in Ghana from multiple perspectives.

## Method

This study examined the following research question from the family perspective: what are the experiences of families in navigating the Ghanaian health system to address the general needs of their children with IDD beyond seeking a diagnosis of IDD? This study took place in Accra, the capital of Ghana. Accra provided an opportunity to explore what may happen to children with IDD within the context of the pinnacle of the Ghanaian health system, especially since research on how the Ghanaian health system addresses the general health needs of the children is sparse. Accra is home to some of the best and renowned health facilities in Ghana. It is estimated that Ghana’s population is 24,658,823, of which 16% resides in Accra (Ghana Statistical Service 2012).

### Design

This study was guided by the interpretive description qualitative design that allowed the researcher to both describe the experiences of families and extrapolate implicit patterns and meanings embedded in the descriptions to add context (Thorne 2016).

### Participants

The researcher recruited the study participants with the assistance of professionals from government and nongovernment organizations who worked closely with children with IDD and their families in Accra. These professionals introduced the researcher to many of the families involved in this study. Families who were willing to participate in the study also linked the researcher to other potential families in their network. The study participants had to meet the following eligibility criteria: (a) be a primary caregiver of a child with IDD; (b) be able to present proof that the child has been diagnosed with IDD by an appropriate health professional (e.g. a clinical psychologist); and (c) be able and willing to participate in semi-structured interviews to gather data for this study.

### Data collection

This study is a component of a large research on healthcare for children with IDD in Ghana approved by the research ethics boards of two universities in Ghana and Canada; namely, the Ethics Committee for the Humanities, University of Ghana (ECH 090 14-15), and the General Research Ethics Board, Queen’s University (GREB TRAQ #: 6014124). Participation in this research was strictly on a voluntary basis. The purpose of the study was explained to the participants and only those who provided informed consent were included in data collection. The participants were also informed of their rights to withdraw their consent at any point during data collection with no obligations.

Data was collected by semi-structured interviews, which were audio recorded with permission from the participants. Although the participants were given the option of being interviewed in English (lingua franca of Ghana) or a local language, all of them voluntarily opted for English. Notwithstanding, the participants commonly interspersed their responses with Ga or Twi, which are two widely spoken local languages in Accra, both in which the researcher and research assistants are fluent. Samples of the interview questions included: (a) tell me about a time you had to access health services for your child; (b) what processes did you undergo at the health facility you accessed before you received care for your child (e.g. how were you processed to be seen by a doctor or a specialist?)?; and (c) what did any health professional (e.g. doctor) do for your child? Due to the semi-structured nature of the interviews, the format of the questions was modified to the context of each interview and further questions emerged from participants’ responses as the interviews progressed (Brinkmann and Kvale 2015). The researcher, who was the primary interviewer, has past experience working with children with IDD and their families in Accra as a special educator and psychologist.

### Data analysis

Constant comparison, an analytical method originally from the grounded theory qualitative tradition, was the means by which the interviews were analyzed. This was because interpretive description draws from this analytical approach (Thorne 2016). As recommended by Charmaz (2014) in performing constant comparison, the data analysis involved constant cycling between initial coding and focused coding. While initial coding involved line-by-line coding of every idea in the data, focused coding comprised synthesizing initial codes to generate emerging themes. The initial and focused codes were compared both within each interview and across all interviews to generate the study findings.

### Rigor

As recommended by Creswell and Miller (2000), the steps taken to improve the rigor of this study included triangulation, audit trail, member checking and peer-debriefing. For example, multiple participants from different demographic backgrounds shared their experiences with similar health facilities and other participants discussed their experiences with different health facilities across Accra (triangulation). Further, the researcher and research assistants kept a record of events they perceived could influence the outcome of the study during data collection (e.g. distractions during interviews) along with analytical decisions made during data analysis; and these were taken into consideration during data interpretation (audit trail). The researcher intermittently summarized the participants’ responses to them during interviews to confirm that the meaning the researcher ascribed to the responses represented the true intent of the participants (member checking). As well, the data collection and analysis procedures were audited by a team of scholars in the field of qualitative methods and disability research in low-and middle-income countries (peer-debriefing).

## Results

A total of 22 primary caregivers of children with IDD were interviewed in this study. The average duration of the interviews was about one hour. One interview involved both parents of the child. The demographic characteristics of the primary caregivers (hereinafter referred to as families) and their children are outlined in Tables 1 and 2. The families reported on a variety of needs for which they accessed healthcare for their children in Accra. These needs included: dental care (e.g. tooth extraction), gastrointestinal problems (e.g. constipation, celiac, cholera, persistent vomiting and appendicitis), cardiovascular problems (e.g. hole in heart), vision problems, hearing problems (e.g. otitis media and hearing loss), respiratory problems (e.g. tuberculosis and unknown breathing problems), orthopedic problems (e.g. arthritis), neurological problems (e.g. seizures), poisoning, injuries, typhoid, itchy buttocks, fever, headache, malaria, suspected urinary tract infection, yeast infection and disability management (including medical reviews and therapeutic services).

**Table 1.** Socio-demographic characteristics of participants.

Socio-demographic characteristics		Number of primary caregivers (*n* = 22)^a^
Gender	Male	3
	Female	19
Marital status	Never married	1
	Married	18
	Divorced/separated	1
	Widowed	2
Highest Education	Primary	2
	Secondary	5
	Post-secondary	12
	Unknown	3
Employment status	Employed	16
	Unemployed	5
	Retirement	1
Number of people in household	2-4	7
	5-9	12
	10	1
	17	1
	Unknown	1
Insurance status	National health insurance	18
	Private insurance	8^b^
	No insurance	2

Note:

^a^ Two primary caregivers were from the same family and interviewed together.

^b^ Six primary caregivers had both private insurance and national health insurance.

**Table 2.** Demographic characteristics of the participants’ children.

Demographic characteristics		Gender distribution		Total number of children (*n* = 22)^a^
		Male (*n* = 11)	Female (*n* = 11)	
Type of IDD	Intellectual disability (not comorbid with other specified disabilities or genetic syndromes)	1	1	2
	Down syndrome	1	2	3
	Autism	4	5	9
	Cerebral palsy (with cognitive impairment)	2	1	3
	Seizure disorders	2	3	5^b^
	Global developmental delay	1	–	1
Age range (years)	3-6	4	2	6
	7-10	4	1	5
	11-14	2	6	8
	15-18	1	2	3

Note:

^a^ Two of the 22 children belonged to one family.

^b^ One child with seizure disorders also has cerebral palsy.

Three overarching themes emerged from the data analysis: entry into the health system; consultation with health professionals; and service coordination.

### Entry into the health system

This theme focuses on the point at which the families entered into the health system. This theme included the following sub-themes: types of health facilities accessed and cost; service denial; and wait times.

#### Types of health facilities accessed and cost

All but two of the families revealed that they had accessed healthcare for their children at least once within six months prior to participating in this study. Almost all the families reported that they had accessed a variety of public facilities for their children, which included polyclinics, teaching and pediatric health facilities. Some families reported that they have also accessed private and quasi-governmental facilities for their children. The majority of the families reported that they had national health insurance coverage for their children (Table 1). However, they added that out-of-pocket payment was common: ‘consultation is free once you are having the national health insurance card. And taking [processing] the [hospital entry] card is free… . [But] the health insurance does not cover all the drugs. So when I am going to the hospital I go well prepared knowing very well that there could be some [out-of-pocket] payments to be made’ (Pt T).

#### Service denial

Some families noted that their children were denied services at some health facilities, either because the children had difficulties following entry processing protocols or physicians were unwilling to address the children’s needs: ‘they needed to take her temperature and she wouldn’t allow them to touch her. She wouldn’t allow you to touch her because of her sensory issues. And so the woman [nurse] said that she will not allow us to see the doctor without having her temperature taken. So we just left, we had to leave there without being attended to’ (Pt K). Another family recalled: ‘the doctor we met told us that he came for night shift so we have to wait for the one coming for morning shift to report… . For about two hours, we were still waiting and nobody did anything for us. They only took his temperature and we just kept waiting. So we took his folder out and went to a different hospital’ (Pt Y).

#### Wait times

The wait time to see a physician at the health facilities that provided services for the children was usually between 30 minutes to two hours for non-emergency cases. The families explained that some hospital staff who became aware of the children’s disabilities provided faster services for the children: ‘I told the nurses that my daughter has autism so they didn’t let me join the queue. They just did everything fast for me to see the doctor. [On the contrary,] if you go to some hospitals and if the nurses see the way the child is behaving [behavioral problems] and you talk to them, they will tell you that they can’t help you, you need to join the queue’ (Pt B). Some families were at times able to access faster services through their social connections. On the other hand, there were several instances where families had to wait longer for services, which they attributed to shortage of health professionals to meet high volumes of service demand. The families also explained that there were times that other clients who were behind in the queue were served first: ‘the nurses started calling some people who came after me to see the doctor first before calling me. So I ask the nurses why and they told me “you have to wait, your children are like this [have IDD]. The doctor has to have time for your children and that’s why we are calling other people first before calling you.” But I went there early because I wanted to be the first to be seen’ (Pt Q).

### Consultation with health professionals

The families reported on a variety of health professionals who attended to the children. These professionals included phlebotomists, dentists, speech therapists, physiotherapists, psychiatrists, psychologists, radiologists, ophthalmologists, audiologists, pediatricians, nurses, general medical practitioners and surgeons. The families revealed that the professionals often followed general clinical routines during consultation (e.g. asking about the chief complaint, history taking, physical examination and laboratory testing or imaging request if applicable). Although the professionals normally attempted to speak directly to the children about the purpose of the visit, the families pointed out that they often responded to the professionals on behalf of the children concerning the purpose of the visit: ‘the doctor receives her well and asks her “give me five”; “what’s your name?”; “how old are you?”; “what’s the name of your school?” and she will be answering them all. Then the doctor will ask her what brings us here; “[name of child] what is wrong with you?” So here, I will have to come in because [name of child] will not be able to say it’ (Pt T).

#### Challenges in care administration

There were many times that the professionals experienced difficulties gaining the cooperation of the children during service provision, which families linked to sensory issues that the children had. Further, the families thought that some of the difficulties the professionals experienced were due to the functional and behavioral challenges associated with the IDD of the children: ‘he did not understand what was happening. I think he saw it as a threat. If you are trying to restrain him, he will fight back. And you can’t talk to him to agree’ (Pt O). The families also believed that their children’s association of past unpleasant visits with current visits was responsible for some of the difficulties the professionals faced: ‘they brought him to the recovery room and when he was coming out [of sedation] that’s when he realized that “I don’t want to be here, get me out of here.” When he came out he realized “oh, some years ago when I was in this room it wasn’t that pleasant. Get me out of here”’ (Pt C).

The families explained that the professionals sometimes used play to gain the children’s cooperation. Other times, the professionals resorted to physical restraint. A treatment procedure could be forgone, truncated or substituted with medication if the professionals were unsuccessful in gaining the children’s cooperation: ‘the doctor checked him and said his breathing was not good. So she asked us to go to the emergency [department] so that they will nebulize him. About three to four male nurses [tried to physically restrain him but] this guy was too strong for them so they couldn’t nebulize him… . Because of that the doctor wrote medication for us to go and buy’ (Pt Z).

#### Attitudes of health professionals

While the families perceived that some professionals treated children with IDD in the same ordinary way that those professionals normally treated other clients, other professionals made additional efforts to make the children feel more comfortable:

The dentist carried my boy from the car when we arrived; he was talking to the boy and reassured the boy that he would be fine. Even though the dentist probably knew that the boy wouldn’t understand what he was saying to him, he still talked to the boy, put him in the chair and sat with him for a bit. After that, he and his assistant managed to get some kind of a clasp thing into the boy’s mouth which kept it open for them to deal with the tooth. The next day, the dentist called us to find out if the boy was fine (Pt L).

The families reported on some disturbing attitudes they experienced from some of the professionals. These attitudes included staring at the children and their families, and talking impolitely to the families: ‘one doctor told me at [name of hospital] − my child was sick and I took him there − the doctor said: “once a Down syndrome, always a Down syndrome.” And the way he put the thing, you know, it’s like he was insulting me’ (Pt Z). Another family reported:

I’m a nurse myself and most of the nurses [at the facilities I visit] know that. So sometimes, they’re quite okay. But there are other nurses who just wouldn’t mind at all that I’m also a nurse and they will be shouting at me: ‘why are you not restraining the child? “*Maame, shwɛ wo ba no*”; I mean, “woman, control your child” that kind of thing… . I took him for speech therapy and before the therapist gathered her data, I told her that my boy is hyperactive and all that. And then during the session, the therapist was like “you are a very stubborn boy, sit down” and then she took a cane and said “if you do not sit down, I will cane you”. [She said] “make sure you control the child” and she was just shouting at me. Since then I decided that I am not going for that anymore (Pt Y).

These disturbing attitudes sometimes broke the hearts of the families: ‘the way the woman was talking to me, I couldn’t have the chance to even explain that my daughter has autism or something. So the thing was so painful that I had to even shed tears with my daughter and go away – I left the hospital that day to another hospital. Yes, it was very very painful’ (Pt B). Some families reported that they were able to clearly express their displeasure to the professionals. However, other families perceived that the professionals had more power, which made it difficult for the families to take any meaningful actions against the professionals:

Because we are in their hands if we say anything back maybe they will not take it lightly – maybe they will say something that will embarrass you in the presence of everyone, so what would you do? They will put your folder aside and take care of other patients because you made them know that they should not be talking to you in a disrespectful way. They will not attend to your child [early]; they will do what they feel like doing before they will attend to the child (Pt G).

### Service coordination

Services were delivered in a seamless fashion in some of the health facilities that the families accessed: ‘his blood was taken [for laboratory testing] before we went to see the doctor. When the lab result arrived, the medicine they had to administer also came with it’ (Pt R). However, many facilities did not offer seamless services and in some cases, the families were asked to get services not available at the facility elsewhere on their own: ‘the doctor said I should go and buy bandage or something [they needed for treatment]. I didn’t get some at [name of hospital accessed]. So I went to town to get it but many pharmacies did not have it. I eventually found one to buy and it was very expensive’ (Pt V). Further, the infrastructure at some health facilities was in poor state:

After the surgery, [name of child] was on the stretcher and we brought him with the ambulance [to the recovery ward]. We had about 4 or 5 kids in a small ambulance with no AC and everything. My child couldn’t sit so he had to be on the stretcher. And the other kids were with their parents in the ambulance too. My child and I sat in the ambulance for a while before they brought in the other children and their parents… . That five or three minutes’ drive wasn’t pleasant. The ambulance is supposed to take one person. We were crowded in the thing and also the heat. We brought him with the ambulance and they had to carry him on the stairs like that, the elevator wasn’t working (Pt C).

## Discussion

Despite the growing literature on the health needs of children with IDD and their experiences in accessing healthcare in many parts of the world, relatively little is known in the Ghanaian context (Adugna *et al*. 2020, Magnusson *et al*. 2019). This study explored the experiences of families in navigating the Ghanaian health system to address the general health needs of their children with IDD. Similar to previous research linked to the screening and diagnosis of IDD in Ghana (Bello *et al*. 2013, Kyeremateng *et al*. 2019), the study findings illustrate that there is a variety of public and private health facilities that families can access for their children, especially in Accra. However, the study findings highlight that some of the facilities may refuse to provide services if the children have difficulties following entry processing protocols. This implies that not all facilities adapt their services to provide equitable access for children with IDD. Instead, the onus of finding a means to fitting into existing service provision systems lies on the children and their families. Meanwhile, children with IDD may have difficulties following health administration protocols because of the neurological, behavioral and sensory issues that may be associated with IDD (American Psychiatric Association 2013). Not having flexible service provision systems to accommodate the needs of children with IDD compromises the children’s fundamental rights to equitable healthcare (Márton *et al*. 2013, Unicef 1989). There is a need to assess the capacities of health facilities to adapt their services to the needs of children with IDD and strengthen those capacities. For example, if the capacity assessment highlights that health professionals lack adequate knowledge and skills to meet the children’s needs, a training program could be designed to equip the professionals with relevant knowledge and skills. However, the extent to which the professionals can apply relevant knowledge and skills can be limited should there be a lack of appropriate resources and infrastructure to support the application of the knowledge and skills.

The study findings show that Ghana’s national health insurance makes it possible for families to access healthcare services for children with IDD to some extent. Research shows that the national health insurance may have improved access to healthcare for women and children in Ghana (Mensah *et al*. 2010, Singh *et al*. 2015). However, the families in this study highlighted that the insurance does not cover all services that may be required by the children; hence, families who may not be able to afford to pay out-of-pocket for those services not covered by any insurance they have may struggle to secure adequate healthcare for their children. Although studies show that the national health insurance may not also cover all services for typically developing children (Abuosi *et al*. 2015), IDD may be associated with greater need of health services to manage the disability itself and health issues that may be linked to the disability (O’Hara *et al*. 2010, Taggart and Cousins 2014, Wehmeyer *et al*. 2017).

The families in this study revealed that some health professionals who became aware of the children’s disabilities provided faster services at times for the children. Further research is needed to understand more in-depth what informs the professionals’ decisions on whether or not to offer the children faster services. For example, while attending to other clients who are behind in the queue before attending to children with IDD on the premise that it may take a longer time to address the children’s needs, as reported by some of the families in this study, may amount to discrimination, the study findings also suggest that this may be a strategy employed by the professionals to create enough time for the children without feeling pressured to rush through consultation because of high volumes of service demand. Faster services may not necessarily lead to efficient service provision if the professionals do not have ample time to deliver (Sullivan *et al*. 2018). On the other hand, asking the families to wait longer to enable the professionals to make adequate time for the children may lead to delayed care.

The study findings show that some health professionals may use play or physical restraint to successfully work around functional and behavioral challenges that the children may have during service delivery. However, consistent with the findings of a systematic review that indicates health professionals may have difficulties dealing with the functional and behavior challenges that may be associated with IDD (Iacono *et al*. 2014), the study findings also highlight that professionals in Ghana may not always be successful in managing these difficulties to the extent that they may have to forgo some recommended treatment if necessary. While forgoing recommended treatments may greatly put the children at risk, medical ethics may allow the professionals to do so if they lack the necessary infrastructure and resources to adequately guarantee the safety of their clients and everyone involved in providing care (British Medical Association 2012, Laurie and Dove 2019). It is therefore important for health administrators and policymakers in the country to ensure that health facilities have the necessary infrastructural support to provide safe services for children with IDD. In the meantime, it is wise to improve the ability of health providers to optimally use the resources available to them in addressing the needs of children with IDD.

The study findings demonstrate that children with IDD and their families may experience mixed attitudes from health professionals in Ghana. These findings are similar to a Canadian study that reported mixed attitudes of health professionals toward children with autism and their families (Muskat *et al*. 2015). Although research shows that families of children with IDD in Ghana and people with IDD in other parts of the world may experience challenging attitudes from health professionals (Iacono *et al*. 2014, Oti-Boadi 2017), the study findings show that there may be some positive attitudes to an extent. In addition to accommodating the needs of children with IDD as far as possible, it was encouraging to note that the families involved in this study reported that some professionals attempted to speak to children with IDD directly during consultation. While it can be a challenge for some children with IDD to understand health professionals, even in layman’s language (American Psychiatric Association 2013, Espinoza and Heaton 2016), involving and speaking directly to the children during consultation is a recommended practice (Sullivan *et al*. 2018). However, it is critical that health professionals present information at the children’s level of understanding (Perry *et al*. 2014).

Not all the attitudes experienced by the families and their children from the professionals were positive. These study findings are consistent with other research findings both in Ghana and across the world (Iacono *et al*. 2014, Oti-Boadi 2017). The literature shows that it is common for people with disabilities in Ghana to experience attitudinal barriers in society on the basis of sociocultural beliefs that associate disabilities with evil (e.g. curse or demonic possession) (Mensah *et al*. 2008, Slikker 2009). As well, health professionals in Ghana are notorious for losing their temper with clients regardless of disability status (Andersen 2004, Mannava *et al*. 2015, Moyer *et al*. 2014, Yakong *et al*. 2010). Andersen (2004) indicates that challenging attitudes from the professionals in Ghana may be induced by stress from poor working conditions. Heated disputes among the professionals in Ghana over who is expected to perform certain clinical duties (e.g. anesthesia) may also translate into unprofessionalism toward clients (Aberese-Ako *et al*. 2015). The study findings demonstrate that challenging attitudes from health providers toward children with IDD and their families can impede healthcare for the children by discouraging families from accessing services for their children. Research is needed to thoroughly understand the differentiating factors between the providers in the country who show positive attitudes toward children with IDD and their families, and the providers who show less positive attitudes; which can provide health administrators and policymakers with critical directions for improving providers’ attitudes toward the children and their families. In the meantime, encouraging client-centred healthcare delivery has the potential of leveling the power dynamics that exist between health providers and their clients (Constand *et al*. 2014), especially since some of the families involved in this study did not think that they had enough power to hold health providers accountable. Client-centred healthcare delivery can improve the ability of both health providers and their clients to equally hold one another accountable and promote mutual respect (Constand *et al*. 2014).

People with IDD may have challenges navigating the complexity of health systems (Ali *et al*. 2013). It is encouraging that the families in this study reported that service coordination in some of the facilities they accessed for their children was seamless. However, this was not the case in all facilities that they accessed. More research is needed to better understand the characteristics that distinguish between the facilities that provide seamless services and those that do not. Meanwhile, the practice of making families responsible for providing essential services not available at health facilities experienced by some of the families in this study may put the children at risk, especially if the families are not able to provide those services in a timely manner. Notably, this practice may also be experienced by other clients without IDD (Morgan *et al*. 2018). Further, the study findings illustrate that some health facilities in Accra may have poor infrastructure, which can compromise the quality of healthcare. For example, putting a child who has just gone through surgery on a crowded ambulance, as described by some of the families in this study, can increase the risk of infection among passengers. Meanwhile, it is possible that health providers may be caught between denying many clients services because of limited resources and serving as many clients as possible who are in need of the limited resources available. Typically, many African countries, including Ghana, lack adequate funding for health and other development projects (Beegle and Christiaensen 2019). In addition, policymakers and administrators in Ghana may lack the political will to provide the necessary amenities and services that will promote health and prosperity for all (Abukari *et al*. 2015). It is crucial that policymakers and administrators make judicious use of resources that are available to them to provide services for everyone in need.

A number of study limitations are worth mentioning. Although it may be hypothesized from the study findings that the issues contributing to inequitable healthcare for children with IDD in Accra may be more severe in rural areas, more research is needed to provide a better understanding of the rural context. Further, while this study provides some preliminary insights on the experiences of families in addressing the needs of their children with IDD within the Ghanaian health system, it is only based on the family perspective. Exploring the perspectives of health professionals, and health system organization and administration can add to the context of the experiences described by the families in the health system. For example, one of the families in this study reported an incident in which physicians refused to treat her child on the basis that their work shift was over. In this incident, there could be a deeper rationale best known to the physicians for their actions (e.g. terms of employment and overtime compensation issues). Further, information on the measures in place at the facility where this incident happened to ensure that physicians took over their shifts swiftly or remained at post until they had officially handed over to their colleagues were unknown. Although the researcher and research assistants made concerted efforts to recruit participants of diverse demographic backgrounds, most of the participants obtained were married females with post-secondary education and employment. More research is needed to better understand the perspectives of fathers, the experiences of children with IDD from single parent homes and the experiences of children with IDD having unemployed parents or parents with minimal education. While the researcher’s past experience working with children with IDD and their families in Accra as a special educator and psychologist enhanced the researcher’s ability to build rapport with participants during interviews, there is also the potential that an interview conducted by a lay person or another parent of a child with IDD may have elicited different responses than one conducted by a professional who is not a parent of a child with IDD.

## Conclusion

This study provides a preliminary understanding of the experiences of families in addressing the general health needs of their children with IDD within the Ghanaian health system aside from disability screening and diagnosis, particularly since research in this regard is sparse. The study findings demonstrate that the experiences of families and their children with IDD in the Ghanaian health system may be mixed. Specifically, while families can have positive experiences within the Ghanaian health system, especially in Accra, as far as addressing the general health needs of their children with IDD is concerned; families can also confront complex challenges within the health system that may undermine healthcare for their children to an extent.
